# Supplementary material for: Puzzles in modern biology. III.Two kinds of causality in age-related disease
Source: F1000Res. 2017 Jan 10;5:2533. Originally published 2016 Oct 18. [Version 2] doi: 10.12688/f1000research.9789.2 (PMC5288673; doi:10.12688/f1000research.9789.2)
Supplement: Peer review history

# Peer review: Referee response for version 2

**DOI:** 10.5256/f1000research.11457.r19508

**Published:** 2017-01-27

**Authors:** Marta Bertolaso

**Affiliations:** 1 Institute of Philosophy of Scientific and Technological Practice, Faculty of Engineering, University Campus Bio-Medico Rome, Italy, Rome, Italy

I thank the author for taking my suggestions into account. The added remarks, as the author rightfully notes, are included as part of the final publication. I think those responses are useful in contextualizing and explicating what methodology is being employed to perform what philosophers call an “explanatory reduction” of cancer, i.e., the methodology that gives “rate” a meaning, and within which we must distinguish rate and function. The response now provides a particularly clear illustration of how reduction proceeds in isolating individual processes that influence rate “within the background of many other often unidentified processes that must also be acting” (see Frank 2007). Therefore, despite the fact that version 2 of the paper did not change very much with respect to version 1, I approve the publication.

 Like the author, I am convinced that the evolutionary perspective on cancer here adopted will need to be put into dialogue with organism-centered dynamic views, focused on the dynamic stability (and loss thereof) of tissues, organs, and organisms. Indeed, I observe that the whole field of cancer research is moving towards more adequate definitions of cancer that focus on explanatory levels that are increasingly appropriate to the disease. Experimental and modeling works, and well as rigorous methodologies like those defined by Frank, are not diminished, rather, they find a context and a proper place that justify their explanatory power, as well and their limits and validity conditions.

I have read this submission. I believe that I have an appropriate level of expertise to confirm that it is of an acceptable scientific standard.

## References

[1] FrankSA: Dynamics of Cancer: Incidence, Inheritance, and Evolution.2007; 20821846

# Peer review: Referee response for version 1

**DOI:** 10.5256/f1000research.10554.r18447

**Published:** 2017-01-03

**Authors:** Marta Bertolaso

**Affiliations:** 1 Institute of Philosophy of Scientific and Technological Practice, Faculty of Engineering, University Campus Bio-Medico Rome, Italy, Rome, Italy

Reading the title of this paper, I have at least two questions to pose to the author: first, what are ‘age-related diseases’ and how does cancer qualify as such; second, what is the puzzle here. As for the second question, I don’t find in the paper any puzzle. Instead, what I find is a pretty clear exposition of two different approaches to finding and evaluating causal factors. Four arguments and a final suggestion follow.

  

1. Although philosophers would be more demanding in using terms such as “function” and “kinds of causality”, I think Frank’s distinction between rate and function can be useful and bring clarity in the following domains: setting up experiments, interpreting results, and making mathematical models. In the paper, the section entitled “Identifying causal factors” is the most illustrative under this respect, the rest of the paper lacking a bit of context for the reader to understand the importance of what is being argued.
2. About age-related diseases, I would not think of cancer primarily as an age-related disease, and for good reasons I think, although I agree that there are important theoretical links. Cancer is one of those diseases that include alterations of the dynamic stability of the organism. Age is not enough, not essential, although, of course, it increases the risk. Therefore, the role that the emphasis on cancer as an age-related disease plays in this paper should be clarified. Rather, cancer may be more properly defined as a life history related disease (indeed, it might help us in reconceptualising aging itself). I think this is a key feature that Frank captures in his distinction between “the physiological function itself” and “the processes that change physiological function”. These latter processes have to do with the dynamic stability of the organism. If the author wants to stress this point, in my view, it would be more useful to talk about “the modified processes that don’t stabilize function anymore”, therefore letting new functions emerge. This way of expressing would be no slight difference, since it would adopt an organism centered perspective as opposed to a cancer centered one (centered on genetic mutations in individual cells and cell populations). The author’s own distinction, reformulated as a distinction between ‘function change’ and ‘process change’, would then be more sounding and would express all its usefulness to understand scientific practice.
3. The author seems to be adopting a strict selectionist perspective (cancer is an evolutionary process of discovery). It is in this perspective that “rate” makes sense: it is the rate of an ongoing process of discovery of particular biological functions that are necessary for cancer. The cancer centered selectionist perspective imbues the language of prevention, leading to paradoxical statements such as saying that wound healing, cell division, epigenetic instability, or increased mutagenesis are “physiologically unimportant”. Yet, several available interpretations and models of cancer, both in the lab and in the clinic, seem to suggest a different preventive and therapeutic approach, one that empowers the organism and helps it to find new stable states. In a recent book (Bertolaso 2016) I have reviewed some of this territory and argued for a more articulated perspective that recomposes some paradoxes.
4. Frank’s reductionist approach seems useful for the methodological purposes mentioned above, but it might be misleading if adopted as a general approach to carcinogenesis and cancer onset. “Basic understanding of onset”, as it is expressed here, seems to be a rather impossible goal: we should find all the factors that influence rate (also by using factors functions as a clue) and then study their influence “within a particular background of other rate processes” and “within the complex interacting ensemble of processes that determine the overall rate of onset”. This seems to be the kind of approach of ‘getting complexity by aggregation’ that has been declared as desperate by Weinberg (2014). I would make clearer that the selectionist perspective (with the associated ‘rate’ talk) is one possible useful reduction of a disease that is probably best seen as affecting a tissue, an organ or an organism and their dynamic stability and stabilizing functionalities.

 

 In summary, I would suggest the author to reframe and clarify the structure of the paper so as to make clear its scope: i.e., proposing a clear distinction (not a puzzle) that must be taken into account in modeling and experiment. I would add a little bit of background to emphasize this importance. Comments in n.2 can be of help although the wider perspective I am suggesting is not strictly needed if the methodological approach and the explanatory import of the experimental context of this paper is adequately narrowed down.

I have read this submission. I believe that I have an appropriate level of expertise to confirm that it is of an acceptable scientific standard, however I have significant reservations, as outlined above.

## References

[1] WeinbergRA: Coming full circle-from endless complexity to simplicity and back again. *Cell*.2014;157(1) : 10.1016/j.cell.2014.03.004 267-71 10.1016/j.cell.2014.03.004 24679541

[2] BertolasoM: Philosophy of Cancer.2016;18: 10.1007/978-94-024-0865-2 10.1007/978-94-024-0865-2

# Response

**Published:** 2017-01-06

**Authors:** Steven Frank

**Affiliations:** Department of Ecology & Evolutionary Biology, University of California, Irvine, USA

I thank Marta Bertolaso for her many thoughtful comments that extend the scope of the discussion. This exchange is included as part of the final publication of my article, so I confine most of my response to these comments. In a few cases, I note revisions in Version 2 that address some issues. 

 Italics quote from Bertolaso’s review.

*Reading the title of this paper, I have at least two questions to pose to the author: first, what are ‘age-related diseases’ and how does cancer qualify as such; second, what is the puzzle here.*

 “Age-related diseases” are diseases that change with age, implicitly, diseases that change with age in some regular manner. Colorectal cancer incidence increases with age. Retinoblastoma incidence increases very early in life, then the incidence declines to nearly zero after age five or so. Both diseases are related to age.

 With regard to the puzzle here, the abstract states: “The rate-function duality provides the basis for solving puzzles of age-related disease.” Thus, I agree that this article does not pose a puzzle, but rather provides some tools that can help in solving puzzles. The article contains a section with the title “Solving different puzzles” that emphasizes this goal. In my revision, I have also added a final section “Prospect” to further emphasize that point. 

*In the paper, the section entitled “Identifying causal factors” is the most illustrative under this respect, the rest of the paper lacking a bit of context for the reader to understand the importance of what is being argued.*

 F1000Research has alternative word limits that set the kind of article and the associated open access fees. I wrote this article to fit within the limit of 1000 words, leading to brevity. To provide examples, I coupled this article with a following article in this series, which includes discussion of neurodegeneration, cancer and heart disease (http://dx.doi.org/10.12688/f1000research.9790.1). In my revision, I have added a final section “Prospect” with a pointer to the following article. My article also cited my extensive summary of cancer in my 2007 book (ref. 5).

*About age-related diseases, I would not think of cancer primarily as an age-related disease, and for good reasons I think, although I agree that there are important theoretical links. Cancer is one of those diseases that include alterations of the dynamic stability of the organism. Age is not enough, not essential, although, of course, it increases the risk. …*

 This comment and the following one raise a stimulating and nuanced view of dynamic stability and disease (see the full text of comments 2 and 3 in Bertolaso’s original review). I am not going to change course and take up that view, because it is not the way in which I was thinking about the problem. I do see the value and appreciate being introduced to this alternative. I think there is an opportunity, in the future, to consider the relative merits of Bertolaso’s framework in relation to my views. Perhaps, over time, there will be a merging of the best aspects of the alternative perspectives into something that will help us to understand these problems more clearly.   

*Frank’s reductionist approach seems useful for the methodological purposes mentioned above, but it might be misleading if adopted as a general approach to carcinogenesis and cancer onset. “Basic understanding of onset”, as it is expressed here, seems to be a rather impossible goal: we should find all the factors that influence rate (also by using factors functions as a clue) and then study their influence “within a particular background of other rate processes” and “within the complex interacting ensemble of processes that determine the overall rate of onset”. This seems to be the kind of approach of ‘getting complexity by aggregation’ that has been declared as desperate by Weinberg (2014).*

 I never suggested that one could find “all the factors that influence rate” or that one should try to do so. My book Dynamics of Cancer (ref 5) discusses at length how to go about studying individual processes that influence rate within the background of many other often unidentified processes that must also be acting. The essential approach combines two aspects. First, one must have a hypothesis about how a particular rate process alters progression, within a conceptual or theoretical framework for disease progression. Second, one must test that hypothesis by perturbing the particular process and observing the change in the age-incidence curve. If one can consistently predict the pattern of change in age-incidence curves with respect to perturbation of hypothesized rate processes, then one is moving in the right direction. This approach is the opposite of “getting complexity by aggregation.” It is instead a method to isolate putative causes in a testable manner. In this way, one can parse apparent complexity by a practical method of simplification, without oversimplifying. 

*In summary, I would suggest the author to reframe and clarify the structure of the paper so as to make clear its scope…*

 My revised Version 2 adds a brief section “Prospect” to clarify the intended scope of the article, a small step in the suggested direction but not a complete reframe of the structure.

# Peer review: Referee response for version 1

**DOI:** 10.5256/f1000research.10554.r17104

**Published:** 2016-11-21

**Authors:** Anya Plutynski

**Affiliations:** 1 Department of Philosophy, Washington University St. Louis, St. Louis, MO, USA

The title is somewhat misleading. Are rate and functional changes genuinely two different "kinds" of causality? How exactly are kinds of causality distinguished?  As a general rule, I'm not a fan of multiplying kinds (whether of causation, or other entities, processes, etc.) without good reason. While the author is right to point out that one can (and should) distinguish how a disruption affects rate of onset versus how disruptions affect specific functions, I'm not entirely sure that this warrants the claim that these are two distinct kinds of causation.

 Also, regarding the general thesis: surely it's true that changes to rate of onset can involve compromises in function (in some sense), and compromises in function can also change rate of onset? Suppose a gene (e.g., BRCA) is associated with genetic stability or appropriate chromosomal division during mitosis. Mutations such a gene can lead to earlier onset of cancer, but surely also mutations to such genes compromise a function (namely, cell division). Surely the two are not altogether independent?

 Other than this major worry, most of my concerns have to do with clarity of expression:

- Some of the explication of key ideas is all too brief, or the writing is a bit unclear, or difficult to understand.  E.g.,“Causal factors of cancer illustrate the duality between rate processes of discovery” - I’m not sure what “rate processes of discovery” means… does the author mean rates of incidence? The causes of rates of actual discovery of a tumor, via screening, or perhaps diagnoses on the basis of symptoms surely include but are not limited to biological causes (e.g., the skill of pathologists, the effectiveness of our screening tools, etc.).  i.e., "rate processes of discovery" is potentially misleading.
- Also the claim that there is a “duality between rate… and necessarily physiological function" is somewhat difficult to interpret….  I think that the author simply means that these two outcomes (rate of onset and functional disruption) are different, and their causes are different as well. Moreover, I think that the author simply means that we ought to be clear about which outcome interests us, and not assume that whenever we affect function, we also affect rate of onset, and vice versa? Is this a common conceptual confusion in the literature? If so, an example or two as illustration would motivate the reader to see this as a serious concern worth policing in future.
- Also, the claim that X or Y functional change is a “necessary cause of tumor development” is somewhat misleading. Few very specific functional changes are "necessary" for cancer, though some may be more important than others. To be sure, some "generic" functional changes are necessary for cancer, but I don't think that the author means to suggest that ONLY IF this particular function were disrupted in this particular way, would cancer eventuate. Many functions are disrupted in a variety of different ways – the same pathway may be compromised in quite different manners.
- I’m also skeptical of claims about the notion of “physiologically necessary function.”  The author claims, “Experimentally, one may reverse a physiological change and measure the abrogation of a cancerous state. Success points to a physiologically necessary function.” Many functions in biological systems are robust exactly because there are duplicated gene or blocks of genes that play similar functions.  E.g., redundancy is a common feature of biological systems. So, while we may think of a particular realization of function as "necessary," it's of course possible that when such a function is compromised, another (similar) mechanism could play a similar functional role.

I have read this submission. I believe that I have an appropriate level of expertise to confirm that it is of an acceptable scientific standard.

# Response

**Published:** 2017-01-06

**Authors:** Steven Frank

**Affiliations:** Department of Ecology & Evolutionary Biology, University of California, Irvine, USA

I appreciate Anya Plutynski's thoughtful comments. I understand her critical perspective and agree with many of her specific points. However, in my comments below, I suggest that we may be focusing on different aspects of the problem. Both perspectives are valuable. In that regard, it is very helpful to have this exchange included as part of the published version of the article. 

 Italics quote from Plutynski’s review.

*Are rate and functional changes genuinely two different "kinds" of causality? How exactly are kinds of causality distinguished?  As a general rule, I'm not a fan of multiplying kinds (whether of causation, or other entities, processes, etc.) without good reason. While the author is right to point out that one can (and should) distinguish how a disruption affects rate of onset versus how disruptions affect specific functions, I'm not entirely sure that this warrants the claim that these are two distinct kinds of causation.*

 I appreciate that philosophers have refined understanding of notions such as “causality” and “function.” If I had written for a philosophy journal, I would have taken a different approach or, more likely, I would have collaborated with a philosopher to help in getting things right. 

 In my view, I was simply describing what I have repeatedly encountered in the biological literature. Biologists search for what they think of as the causal basis of disease, without reflecting deeply on what they mean. I addressed only one specific aspect of the difficulties that arise from not giving sufficient thought to the motivating goal. I believe that difficulty hinders progress in biological research. 

 In particular, Plutynski has correctly identified my goal as distinguishing between how a disruption affects rate of onset versus how disruptions affect specific functions. I had in mind biologically trained readers. I chose a language that I believe will communicate most effectively with those readers. I welcome this exchange as an addendum that helps to cross the language divide between biology and philosophy, a very useful step for both sides.

*Also, regarding the general thesis: surely it's true that changes to rate of onset can involve compromises in function (in some sense), and compromises in function can also change rate of onset? Suppose a gene (e.g., BRCA) is associated with genetic stability or appropriate chromosomal division during mitosis. Mutations such a gene can lead to earlier onset of cancer, but surely also mutations to such genes compromise a function (namely, cell division). Surely the two are not altogether independent?*

 I agree. The first sentence of my abstract is: “The two primary causal dimensions of age-related disease are rate and function.” A two-dimensional space does not imply exclusivity. Rather, it provides a way to locate a factor simultaneously with respect to the two aspects. Later in the article I say: “The duality of rate and function recur. Each causal factor must be evaluated simultaneously in two dimensions. How does a causal factor alter the rate of tumor development? How does a causal factor alter the physiological function of the tumor?”

*Some of the explication of key ideas is all too brief, or the writing is a bit unclear, or difficult to understand.  E.g.,“Causal factors of cancer illustrate the duality between rate processes of discovery” - I’m not sure what “rate processes of discovery” means… does the author mean rates of incidence? The causes of rates of actual discovery of a tumor, via screening, or perhaps diagnoses on the basis of symptoms surely include but are not limited to biological causes (e.g., the skill of pathologists, the effectiveness of our screening tools, etc.).  i.e., "rate processes of discovery" is potentially misleading.*

 F1000Research has alternative word limits that set the kind of article and the associated open access fees. I wrote this article to fit within the limit of 1000 words, leading to brevity. To provide examples, I coupled this article with a following article in this series, which includes discussion of neurodegeneration, cancer and heart disease (http://dx.doi.org/10.12688/f1000research.9790.1). In my revision, I have added a final section “Prospect” with a pointer to the following article. My article also cited my extensive summary of cancer in my 2007 book (ref. 5).

*Also the claim that there is a “duality between rate… and necessarily physiological function" is somewhat difficult to interpret….  I think that the author simply means that these two outcomes (rate of onset and functional disruption) are different, and their causes are different as well. Moreover, I think that the author simply means that we ought to be clear about which outcome interests us, and not assume that whenever we affect function, we also affect rate of onset, and vice versa?*

 Correct.

*Is this a common conceptual confusion in the literature?*

 Yes.

*If so, an example or two as illustration would motivate the reader to see this as a serious concern worth policing in future.*

 Because of the length restriction and the following article noted above, this article is designed only to sketch the problem in the briefest manner. The following article (see above) notes some applications, for example, in the section Candidate Mechanisms. 

*Also, the claim that X or Y functional change is a “necessary cause of tumor development” is somewhat misleading. Few very specific functional changes are "necessary" for cancer, though some may be more important than others. To be sure, some "generic" functional changes are necessary for cancer, but I don't think that the author means to suggest that ONLY IF this particular function were disrupted in this particular way, would cancer eventuate. Many functions are disrupted in a variety of different ways – the same pathway may be compromised in quite different manners.*

 I mostly agree. I was trying to express what I see as a common mode of expression in the biological literature. For example, most or perhaps nearly all colorectal tumors seem to have acquired abrogation of apoptosis. My interpretation of the biological literature is that, in this case, abrogation of apoptosis is indeed thought of as a necessary cause of tumor development. Of course, all biologists know that words such as “necessary” or “always” are always wrong, because the fundamental lesson of biology is variability. Nonetheless, I think there remains an implicit sense of thinking this way, and thus I wanted to reflect that thought and redirect it to the duality of rate and function, rather than take on refinements of expression.

*I’m also skeptical of claims about the notion of “physiologically necessary function.”  The author claims, “Experimentally, one may reverse a physiological change and measure the abrogation of a cancerous state. Success points to a physiologically necessary function.” Many functions in biological systems are robust exactly because there are duplicated gene or blocks of genes that play similar functions.  E.g., redundancy is a common feature of biological systems. So, while we may think of a particular realization of function as "necessary," it's of course possible that when such a function is compromised, another (similar) mechanism could play a similar functional role.*

 I said if A leads to B, then success points to a physiologically necessary function, in which A is “reverse a physiological change” and B is “abrogation of a cancerous state.” I have changed my wording in my revision to “success points to a candidate for a physiologically necessary function.” I had meant my original “points to” in exactly this way, in the sense of providing a clue.
